# High cell density sequential batch fermentation for enhanced propionic acid production from glucose and glycerol/glucose mixture using *Acidipropionibacterium acidipropionici*

**DOI:** 10.1186/s12934-024-02366-5

**Published:** 2024-03-26

**Authors:** Tarek Dishisha, Mridul Jain, Rajni Hatti-Kaul

**Affiliations:** 1 Department of Pharmaceutical Microbiology and Immunology, Faculty of Pharmacy, Beni-Suef University, Beni Suef, 62511 Egypt; 2 Division of Biotechnology, Department of Chemistry, Center for Chemistry and Chemical Engineering, P.O. Box 124, 221 00 Lund, Sweden

**Keywords:** Propionic acid fermentation, Glucose, Glycerol, Potato juice, High cell density

## Abstract

**Background:**

Propionic acid fermentation from renewable feedstock suffers from low volumetric productivity and final product concentration, which limits the industrial feasibility of the microbial route. High cell density fermentation techniques overcome these limitations. Here, propionic acid (PA) production from glucose and a crude glycerol/glucose mixture was evaluated using *Acidipropionibacterium acidipropionici*, in high cell density (HCD) batch fermentations with cell recycle. The agro-industrial by-product, heat-treated potato juice, was used as N-source.

**Results:**

Using 40 g/L glucose for nine consecutive batches yielded an average of 18.76 ± 1.34 g/L of PA per batch (0.59 g_PA_/g_Glu_) at a maximum rate of 1.15 g_PA_/L.h, and a maximum biomass of 39.89 g_CDW_/L. Succinic acid (SA) and acetic acid (AA) were obtained as major by-products and the mass ratio of PA:SA:AA was 100:23:25. When a crude glycerol/glucose mixture (60 g/L:30 g/L) was used for 6 consecutive batches with cell recycle, an average of 35.36 ± 2.17 g/L of PA was obtained per batch (0.51 g_PA_/g_C-source_) at a maximum rate of 0.35 g/L.h, and reaching a maximum biomass concentration of 12.66 g_CDW_/L. The PA:SA:AA mass ratio was 100:29:3. Further addition of 0.75 mg/L biotin as a supplement to the culture medium enhanced the cell growth reaching 21.89 g_CDW_/L, and PA productivity to 0.48 g/L.h, but also doubled AA concentration.

**Conclusion:**

This is the highest reported productivity from glycerol/glucose co-fermentation where majority of the culture medium components comprised industrial by-products (crude glycerol and HTPJ). HCD batch fermentations with cell recycling are promising approaches towards industrialization of the bioprocess.

**Supplementary Information:**

The online version contains supplementary material available at 10.1186/s12934-024-02366-5.

## Background

The rapid growth in the bioproducts markets and the need to valorize waste streams are major drivers for establishing different biorefineries for the production of platform chemicals from biomass (waste-to-treasure) [1, 2]. Propionic acid (PA) is a three-carbon carboxylic acid with antibacterial and antifungal activities. It is used to preserve food, feed, and pharmaceuticals and is also a platform for other chemicals and materials in the plastic, perfume, and herbicide industries [3].

The industrial production of PA is mainly through chemical synthesis using ethylene derived from fossil resources as a starting material [4]. The interest in PA production from renewable resources started a century ago [5], while the PA market in 2022 was estimated at 463,250 metric tons and is projected to reach around 603,100 metric tons by 2030 [6]. Propionibacteria, a group of Gram-positive bacteria, can produce PA from different carbon sources [7–9]. Recently, this genus was split to include three novel genera: *Acidipropionibacterium*, *Pseudopropionibacterium,* and *Cutibacterium*, based on their GC content, genome size and sequence, among other parameters [10, 11]. The former includes *Acidipropionibacterium acidipropionici*, the most frequently reported microorganism for PA production.

Propionibacteria can assimilate a variety of C-sources including glycerol, glucose, lactose, sucrose, xylose, and lactate, among others, making them attractive for their ability to use a wide range of cheap and available resources [5, 7, 8, 12–18]. However, the nature and degree of reduction of the C-source can impact cell growth and fermentation kinetics [7]. With most C-sources, succinic acid (SA) and acetic acid (AA) are the main by-products. In contrast, glycerol as a more reduced C-source yields PA with fewer acidic by-products [7, 13, 19–21]. Several studies have examined the combination of glycerol and glucose as C-sources and found that a mass ratio of 2:1 g/g (4 mol/mol) was optimal for PA production with high yield, productivity, and minimal AA by-product [22–25].

Several microorganisms use glucose, an abundant sugar, as the C-source and even glycerol is metabolized by many microorganisms. The glycerol market is expected to grow at a rate of 5.34% between 2023 and 2030 reaching 3.67 Billion USD by 2030 [26]. Glycerol is produced in substantial amounts as a side-product of biodiesel production [27, 28], and is also obtained as a by-product from sugar fermentation during ethanol production by yeast [29]. Although glycerol is an attractive substrate due to its abundance and low cost, the fluctuation of its market prices (1–3 USD/L) and the high demand for pure glycerol in pharmaceuticals and cosmetics could impact the establishment of biorefineries based on glycerol [30]. Nonetheless, glycerol is expected to remain an attractive substrate for some time to come.

Regarding the N-source, propionibacteria can grow on various raw materials such as potato extract, corn steep liquor, whey protein, orange juice, apple pomace extract and casein hydrolysate [14, 31, 32]. Potato juice is obtained as a by-product during potato starch processing. It is rich in nitrogen and mineral salts as well as proteinaceous compounds. The acidification of this juice followed by heat treatment causes the precipitation of the protein fraction and yields heat-treated potato juice (HTPJ) [31, 33]. HTPJ is considered a cheap and available N-source for microbial growth in biorefineries [14, 33, 34].

The strong product inhibition exerted on cell growth and acid production is a major limitation in PA production, which reduces volumetric productivity and inhibits process industrialization. Furthermore, the production of high amounts of other organic acids as by-products adds to the challenge. To address this issue, several approaches have been explored, such as increasing the initial cell density through either cell immobilization [13, 35–39] or recycling [14, 40–44], in situ product removal [9, 15, 22, 45–48], increasing N-source concentration [43, 49], using propionic acid-tolerant mutants [17, 35, 50–52], continuous removal of the resulting acid via continuous operation [13, 40], and genetic engineering to reduce the acidic by-products and increase carbon flux towards PA [17, 24, 53–55].

In the present study, propionic acid production by *Acidipropionibacterium acidipropionici* was evaluated using glucose and a mixture of glucose and crude glycerol as C-sources with HTPJ as an N-source in sequential batch fermentations. This was done with the aim of developing a high cell density process with high product yields, volumetric productivity and low process costs using residual biomass streams and cell recycling.

## Results

### Glucose fermentation in batch mode with cell recycling

**Fig. 1:**
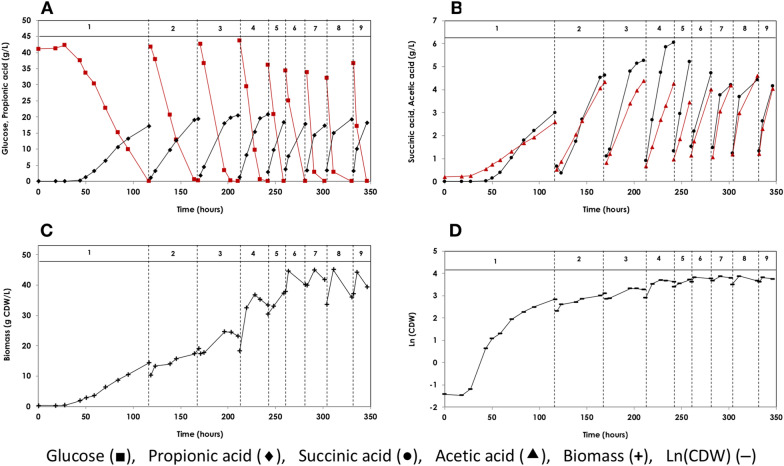
Sequential batch fermentation of glucose for production of propionic acid. Glucose fermentation using *A. acidipropionici* DSM 4900 under sequential batch mode of operation with cell recycling. Fermentation temperature was maintained at 32 °C, pH at 6.5 by the addition of 5N NH_4_OH solution. **A** the concentrations of glucose (black square) and propionic acid (black diamond), **B** the concentration of succinic acid (black circle) and acetic acid (black triangle), **C** the concentration of biomass (**+**), and **D** the microbial growth shown as Ln(CDW) (–). The production medium contained ~ 40 g/L glucose, 0.5 mg/L biotin and 1× heat-treated potato juice

A total of nine consecutive batch fermentations of *A. acidipropionici* with cell recycle were performed over a period of 350 h (~ 14.5 days) using HTPJ (1×), biotin (0.5 mg/L), and glucose (40 g/L) as the production medium (Fig. 1 and Table 1).

**Table 1:** Sequential batch fermentation of glucose using *A. acidipropionici* DSM 4900 with cell recycle

N-source	Batch 1	Batch 2	Batch 3	Batch 4	Batch 5	Batch 6	Batch 7	Batch 8	Batch 9
	HTPJ (1X), 0.5 mg/L biotin								
*Q*_*p*_ (g/L.h)	0.18	0.33	0.55	0.75	1.05	0.76	0.80	0.67	1.15
*Q*_*s*_ (g/L.h)	0.35	0.61	1.08	1.47	2.19	1.68	1.81	1.22	2.57
Y (g/g)	0.53	0.57	0.57	0.55	0.57	0.57	0.57	0.59	0.55
Y (mol/mol)	1.21	1.33	1.24	1.24	1.16	1.10	1.08	1.32	1.09
*r*_*P*_ (g_PA_/g_X_.h)	0.017	0.023	0.033	0.025	0.033	0.022	0.020	0.021	0.028
Initial CDW (g/L)	0.24	10.21	17.42	18.30	30.45	37.84	39.89	33.60	37.19
Final CDW (g/L)	10.69	14.49	16.84	29.57	31.62	35.28	39.89	31.48	40.70
Initial PA (g/L)	0.00	1.13	1.90	1.30	2.88	3.69	3.48	3.45	3.22
Final PA (g/L)	18.01	20.36	21.17	21.27	18.79	18.16	17.93	17.51	18.49
Final AA (g/L)	3.07	4.91	4.73	4.58	3.87	4.11	4.48	4.24	4.36
Final SA (g/L)	3.30	5.05	5.48	6.17	5.36	4.82	4.36	4.05	4.22
PA/AA (mol/mol)	4.75	3.36	3.63	3.76	3.93	3.58	3.24	3.35	3.44
PA:SA:AA (g)	100:18:17	100:25:24	100:26:22	100:29:22	100:29:27	100:27:23	100:24:25	100:23:24	100:23:24

Production of propionic acid from glucose using *A. acidipropionici* DSM 4900 for 9 sequential batches with cell recycling. The concentrations of PA, SA, AA, and biomass, as well as the fermentation kinetics are presented

#### Characteristics of individual batches during sequential glucose fermentation

The first batch was started using 0.24 g_CDW_/L of *A. acidipropionici* cell mass and lasted for 116.5 h (~ 4.9 days) with complete consumption of glucose, resulting in the formation of 18.01 g/L of PA. The batch was characterized by an initial lag phase of 27.5 h (~ 23% of the batch time), where neither glucose consumption nor PA production were observed. Thereafter, the microorganism grew logarithmically at a maximum specific growth rate (*µ*_*max*_) of 0.07 1/h (*t*_*d*_ = 9.9 h) over the following 40 h, before entering the deceleration- and stationary phases. Glucose was utilized simultaneously at a rate of 0.50 g/L.h. Meanwhile, PA production began 45 h after inoculation, with a rate of 0.29 g/L.h until the end of the batch, as shown in Fig. 1. The long initial lag phase resulted in a low final PA volumetric production rate (0.18 g/L.h).

For the second batch, recycled cells from the first batch (10.21 g_CDW_/L) were used to initiate the fermentation process. The fermentation proceeded without a lag phase, and the cells grew logarithmically at a rate of 0.02 1/h (*t*_*d*_ = 43 h). Glucose consumption and PA production started directly after recycling the cells at a rate of 0.93 g/L.h and 0.46 g/L.h, respectively, almost twice those of the first batch. Consequently, the time required for complete glucose consumption was reduced by more than half (only 50.25 h) (Fig. 1).

During the subsequent batches, the initial cell density increased gradually from 17.42 g_CDW_/L in the 3rd batch to 18.3 g_CDW_/L in the 4th batch and remained between 30 and 40 g_CDW_/L until the last batch (from 5 to 9th batch). The time required for complete glucose consumption was reduced to 32 h in the 3rd batch and remained around 19.5 ± 4.16 h for the succeeding batches, representing only 16% of the fermentation time of the first batch. Consequently, the global volumetric glucose consumption- and PA production rates were considerably increased ~ 6.5 times reaching 2.57 g/L.h and 1.15 g/L.h, respectively, in the last batch (Table 1).

PA concentration was steady at 18.69 ± 1.33 g/L throughout the sequential batches (0.56 ± 0.02 g_PA_/g_Glu_). The change in PA concentration as a function of time in each batch was linear, indicating the absence of product inhibition (Fig. 1 and Table 1).

#### By-products from sequential batch fermentation of glucose

SA and AA were the main by-products, with an average concentration of 4.76 ± 0.87 g/L and 4.26 ± 0.55 g/L, respectively, over the entire sequential batches (Fig. 1 and Table 1). In other words, for each 100 g of PA produced, around 50 g acidic by-products were obtained (25 ± 3.25 g SA and 22 ± 2.42 g AA). These acidic by-products represent ~ 1/3 of the total organic acids produced. Carbon dioxide was also released, leading to foam formation within the bioreactor, which was repressed by applying a few drops of anti-foam (polypropylene glycerol).

#### Overall glucose fermentation process kinetics

**Fig. 2:**
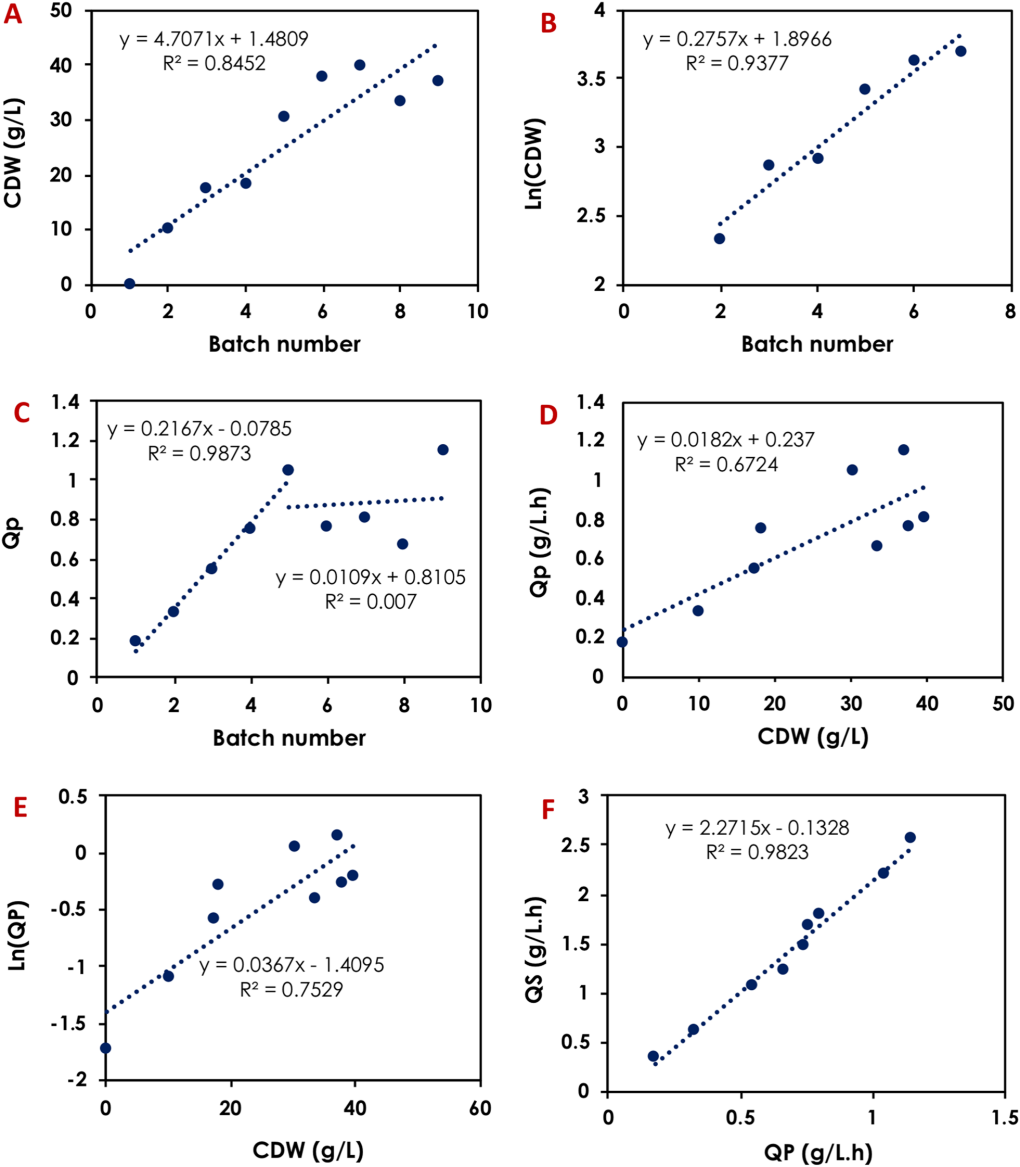
Kinetics of the sequential batch propionic acid fermentation using 40 g/L glucose, 1× HTPJ and 0.5 mg/L biotin using *A. acidipropionici* DSM 4900 cells for 9 sequential batches with cell recycle. The parameters shown are: **A** changes in biomass concentration from batch to batch, **B** changes in Ln(CDW) from batch to batch, **C** changes in volumetric PA production rate (*Q*_*P*_) from batch to batch, **D** changes in propionic acid volumetric productivity (*Q*_*P*_) as a function of initial biomass concentration, **E** Changes in Ln(*Q*_*P*_) as a function of initial biomass concentration for the different batches, and **F** correlation between volumetric glucose consumption rate and volumetric PA production rate

A detailed kinetics analysis revealed that the global PA volumetric productivity increased linearly during the first 5 batches at a rate of 0.22 g/L.h per batch, reaching 1.05 g/L.h in the 5th batch. The productivity dropped slightly during the 6th, 7th, and 8th batches before increasing again to score a maximum of 1.15 in the 9th batch (Fig. 2 and Table 1). The specific PA production rates (*r*_*P*_) followed a similar trend, increasing from 0.017 g_PA_/g_X_.h in the first batch to 0.028 g_PA_/g_X_.h in the last batch (Table 1). The initial cell density was found to have positive correlation with the volumetric productivity (slope = 0.0182), indicating that increasing the initial biomass by 10 g_CDW_/L could result in an increase in PA productivity by ~ 0.18 g/L.h (Fig. 2). Furthermore, the initial biomass was observed to increase logarithmically per batch between batches 2 and 7 (Fig. 2).

**Fig. 3:**
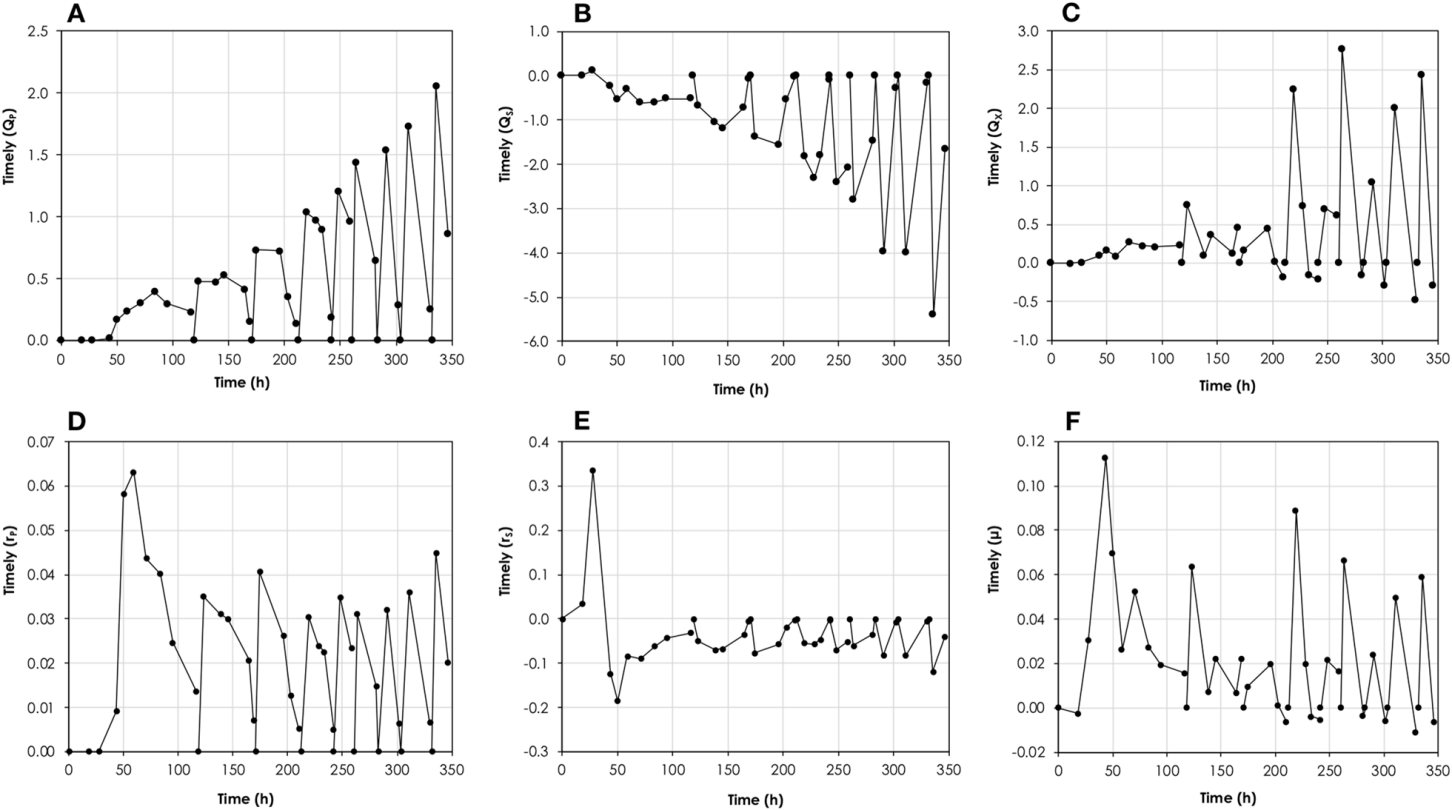
Changes in the volumetric- and specific consumption- and production rates at each sampling point for propionic acid production from glucose using *A. acidipropionici* DSM 4900 growing on 1× HTPJ supplemented with 0.5 mg/L biotin. The fermentation was run as sequential batches with cell recycling. **A** Changes in PA volumetric productivity (g/L.h), **B** changes in volumetric substrate consumption rate (g/L.h), **C** changes in volumetric biomass productivity (g/L.h), **D** changes in PA specific productivity (g_PA_/g_X_.h), **E** changes in substrate specific consumption rate (g_Substrate_/g_X_.h), and **F** changes in specific growth rate (1/h)

Figure 3 shows the changes in the volumetric- and specific consumption- and production rates at each sampling point. During the first batch, the maximum hourly volumetric glucose consumption rate was 0.62 g/L.h, which was increased to 5.40 g/L.h in the last batch. Likewise, the corresponding maximum hourly PA volumetric production rates were increased from 0.39 g/L.h to 2.06 g/L.h, respectively. On the other hand, the hourly maximum specific PA production- and glucose consumption rates were higher during the first batch (0.06 g_PA_/g_X_.h and − 0.18 g_Glu_/g_X_.h, respectively) compared to the succeeding batches (0.04 g_PA_/g_X_.h and − 0.08 g_Glu_/g_X_.h).

### Glycerol/glucose co-fermentation in sequential batch mode with cell recycling

The use of glycerol:glucose mixture has been reported to enhance PA fermentation kinetics [22–25]. Therefore, it was interesting to investigate the performance of this mixture in high cell density (HCD) fermentation.

**Fig. 4:**
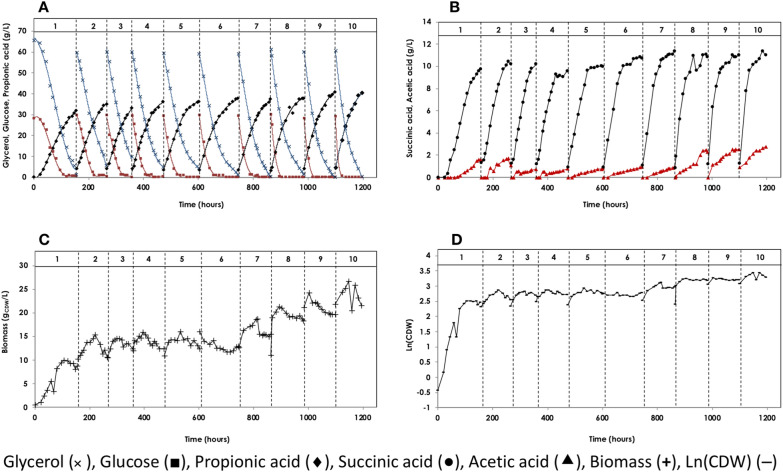
Sequential batch fermentation of glycerol/glucose mixture for production of propionic acid using *A. acidipropionici* DSM 4900 with cell recycle. Fermentation temperature was maintained at 32 °C, pH at 6.5 by the addition of 5N NH_4_OH solution. **A** The concentrations of glucose (black square), glycerol (×) and propionic acid (black diamond), **B** the concentration of succinic acid (Black circle) and acetic acid (black triangle), **C** the concentration of biomass (**+**), and **D** the microbial growth shown as Ln(CDW) (–).The production medium contained ~ 30 g/L glucose, 60 g/L glycerol, and 1.25× heat-treated potato juice. The medium was supplemented with 0.5 mg/L biotin (batch 7), or 0.75 mg/L biotin (batches 8, 9 and 10)

In this study, glycerol obtained from the biodiesel process was used in combination with glucose at a previously reported mass ratio of 2:1 g/g to achieve optimum PA production [21–23, 25]. Sequential batch fermentation was performed for 10 sequential batches over a total period of 1195 h (~ 50 days), with cell recycling using 90 g/L total carbon (60 g/L glycerol and 30 g/L glucose), and a slightly concentrated HTPJ (1.25×) as a nitrogen source. The first 6 batches were run without external biotin supplementation. However, the 7th batch was supplemented with 0.5 mg/L biotin, and the last 3 batches (8th, 9th, and 10th) with 0.75 mg/L biotin (Fig. 4 and Table 2).

**Table 2:** Sequential batch fermentation of glycerol/glucose mixture using *A. acidipropionici* DSM 4900 with cell recycle

N-source	Batch 1	Batch 2	Batch 3	Batch 4	Batch 5	Batch 6	Batch 7	Batch 8	Batch 9	Batch 10
	HTPJ (1.25 X)						HTPJ (1.25 X) + biotin			
*Q*_*p*_ (g/L.h)	0.28	0.36	0.40	0.35	0.33	0.31	0.38	0.39	0.42	0.48
*Q*_*s*_ (g/L.h)	0.59	0.76	0.86	0.70	0.65	0.62	0.76	0.75	0.78	0.93
Y (g/g)	0.48	0.47	0.46	0.50	0.50	0.50	0.50	0.52	0.54	0.51
Y (mol/mol)	0.69	0.71	0.70	0.63	0.75	0.75	0.74	0.78	0.79	0.76
*r*_*P*_ (g_PA_/g_X_.h)	0.032	0.034	0.032	0.030	0.027	0.024	0.024	0.021	0.021	0.032
Initial CDW (g/L)	0.66	10.28	10.54	12.08	10.98	16.07	12.66	11.13	21.23	21.89
Final CDW (g/L)	8.82	10.65	12.44	11.86	12.44	13.03	15.59	18.30	19.69	21.59
Initial PA (g/L)	0	4.09	4.04	4.00	3.37	3.31	3.53	3.20	4.11	4.40
Final PA (g/L)	32.00	34.98	33.00	36.31	36.10	37.68	37.45	37.80	40.70	40.16
Final AA (g/L)	1.59	1.05	0.74	0.78	0.81	0.95	0.93	2.27	2.54	2.75
Final SA (g/L)	9.76	10.222	10.19	9.58	9.98	10.73	11.37	10.83	10.98	11.01
PA/AA (mol/mol)	16.33	27.01	36.33	37.89	36.17	32.28	32.57	13.51	12.99	11.83
PA:SA:AA (g)	100:31:5	100:29:3	100:31:2	100:26:2	100:28:2	100:28:3	100:30:3	100:29:6	100:27:6	100:27:7

Production of propionic acid from glycerol/glucose mixture using *A. acidipropionici* DSM 4900 for 10 sequential batches with cell recycling. The compositions of the culture media (heat-treated potato juice and biotin), the concentrations of PA, SA, AA, and biomass, as well as the fermentation kinetics are presented

#### Characteristics of individual batches during sequential Gly/Glu fermentation

The first batch was initiated using 0.65 g_CDW_/L cells, and no lag phase was observed. The cells grew at a maximum specific growth rate of 0.05 1/h (*t*_*d*_ = 13 h) during the initial 70 h, followed by a short-term deceleration and then stationary phases when the PA concentration exceeded 15 g/L. During the first 20 h, glycerol and glucose consumption were minimal, and subsequently, their consumption rates increased considerably, reaching a maximum of 0.50 g_Gly_/L.h and 0.34 g_Glu_/L.h, respectively (0.84 g/L.h total carbon). Based on these rates, glucose (30 g/L) was consumed until 118 h compared to glycerol (60 g/L), which lasted until the end of the batch (154 h). Only 1.16 g/L of PA was produced during the initial 20 h. Subsequently, the production rate increased reaching 0.33 g/L.h, resulting in a yield of 32.00 g/L PA at the end of the batch.

For the second batch, recycled cells from the first batch (10.28 g_CDW_/L) were used to initiate the fermentation process. The cells grew logarithmically at a rate of 0.01 1/h over the initial 70 h before entering the stationary and decline phases. Glycerol and glucose consumption started immediately after cell recycling, and the glucose assimilation rate was higher (0.48 g/L.h) than that of the 1st batch. Overall, the 2nd batch lasted for 100 h, one-third shorter than the 1st batch. PA was produced simultaneously at a rate of 0.39 g/L.h, reaching a final concentration of 34.98 g/L.

During the subsequent batches (3rd to 6th), cell density was steady at around 12.42 ± 2.52 g_CDW_/L, and consequently, the time required for complete consumption of the entire substrates was stable at around (117.15 ± 19.34 h). Propionic acid concentration over the 6 batches was constant at around 35 ± 2.15 g/L. The PA volumetric productivity was increased from 0.28 g_PA_/L.h in the first batch, reaching 0.35 ± 0.03 g_PA_/L.h over the subsequent 5 batches.

#### By-products from sequential batch fermentation of Gly/Glu

SA and AA were the main by-products obtained at an average concentration of 10.07 ± 0.40 g/L and 0.98 ± 0.32 g/L, respectively, over the first 6 batches (without biotin). In terms of each 100 g PA obtained, 29 g SA and 3 g AA were produced as by-products (PA:SA:AA = 100:29:3). In this case, acidic by-products represented only 24% of the total organic acids produced.

#### Effect of biotin on sequential batch fermentation of Gly/Glu

Increasing the concentration of biotin during the last 4 batches had a noteworthy impact on the PA fermentation pattern. There was a substantial improvement in cell growth, with the initial cell density almost doubling and reaching 21.59 g_CDW_/L in the last two batches. Furthermore, the cells’ ability to completely consume the supplied carbon sources was evidenced by the fast conversion of glycerol near the end of the fermentation, resulting in a shorter fermentation time of 95 h. As a result, the volumetric PA productivity increased from 0.35 ± 0.03 g/L.h during the previous batches to a maximum of 0.47 g/L.h in the last batch. The PA yield was unaffected, indicating that the same total carbon sources are directed toward PA. However, the concentration of AA was increased 2.6 times reaching 2.75 g/L, while that of SA remained unchanged. Consequently, the final PA/AA molar ratio decreased from as high as 37.8 to as low as 11.83 mol_PA_/mol_AA_.

#### Overall Gly/Glu fermentation process kinetics

**Fig. 5:**
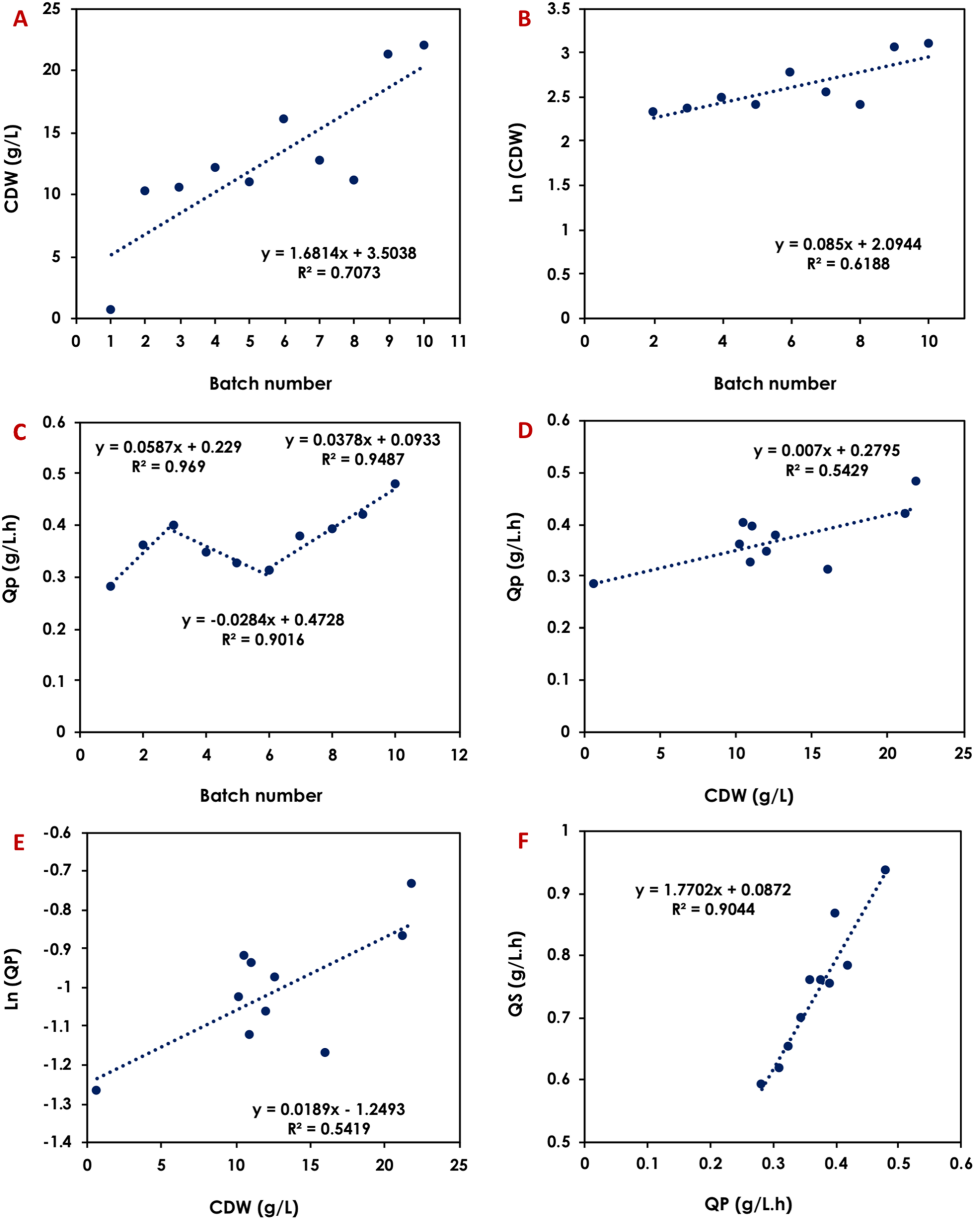
Kinetics of the sequential batch propionic acid fermentation using 30 g/L glucose, 60 g/L crude glycerol, 1.25× HTPJ ± biotin using *A. acidipropionici* DSM 4900 cells for 10 sequential batches with cell recycle. The parameters shown are: **A** changes in biomass concentration from batch to batch, **B** changes in Ln(CDW) from batch to batch, **C** changes in volumetric PA production rates from batch to batch, **D** changes in propionic acid volumetric productivity as a function of initial biomass concentration, **E** Changes in Ln(*Q*_*P*_) as a function of initial biomass concentration, and **F** correlation between volumetric substrate consumption rate and volumetric PA production rate

A detailed kinetics analysis showed that the volumetric productivity increased linearly during the first 3 batches at a rate of 0.059 g/L.h per batch reaching a maximum of 0.40 g/L.h. Subsequently, the volumetric productivity decreased linearly through the subsequent 3 batches at a rate of − 0.0284 g/L.h per batch. However, after the addition of biotin, the volumetric productivity increased again at a rate of 0.332 g/L.h per batch until the end of the fermentation cycles (Fig. 5).

**Fig. 6:**
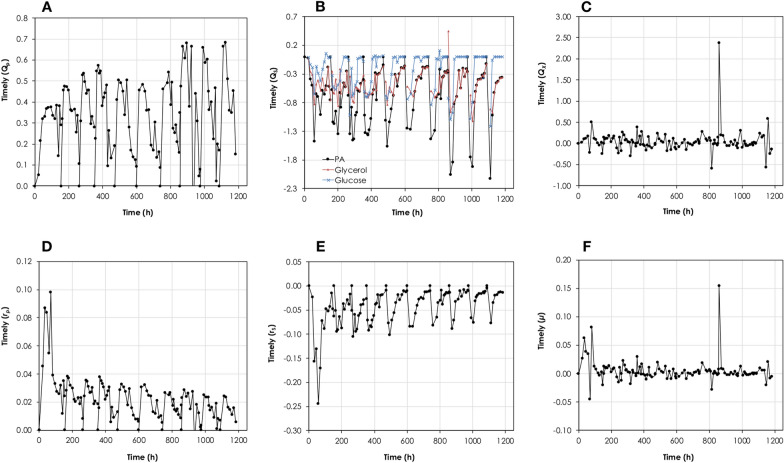
Changes in the volumetric- and specific consumption- and production rates at each sampling point for propionic acid production from glycerol/glucose mixture (90 g/L total C-source) using *A. acidipropionici* DSM 4900 growing on 1.25× HTPJ without- (batches 1–6) and with (batches 7–10) biotin supplementation. The fermentation was run as sequential batches with cell recycling. **A** Changes in PA volumetric productivity (g/L.h), **B** changes in volumetric substrate consumption rate (g/L.h), **C** changes in volumetric biomass productivity (g/L.h), **D** changes in PA specific productivity (g_PA_/g_X_.h), **E** changes in substrate specific consumption rate (g_Substrate_/g_X_.h), and **F** changes in specific growth rate (1/h)

Figure 6 illustrates the changes in the volumetric and specific consumption and production rates calculated for each sample point. The hourly specific substrate consumption and propionic acid production rates were higher during the first batch, reaching a maximum of − 0.24 g_S_/g_X_.h and 0.10 g_PA_/g_X_.h, respectively, compared to the remaining batches. On the other hand, the corresponding maximum hourly volumetric rates were almost equal during the initial 6 batches and then increased during the last three batches, reaching a maximum of − 2.13 g_S_/L.h and 0.68 g_PA_/L.h during the last batch. The propionate yield was stable around 0.50 ± 0.02 g_PA_/g_substrate_. The highest hourly volumetric biomass production rate and maximum specific growth rate were observed during the 8th batch directly after the addition of 0.75 mg/L biotin, where the cells’ concentration increased from 11.13 g_CDW_/L to 20.66 g_CDW_/L within 4 h (2.38 g/L.h) at a maximum specific growth rate of 0.15 1/h (*t*_*d*_ = 4.62 h).

## Discussion

Propionibacteria are capable of utilizing different carbon sources to produce PA as the main end product. To be economically competitive with the chemical route, a PA concentration of about 100 g/L and a volumetric production rate of ~ 2 g/L.h are required [56–58]. High cell density fermentations are one of the useful strategies that ensure PA production at a high rate with high concentration, minimal product inhibition, and minimal risk of contamination.

Although fermentation of glucose and glycerol/glucose mixture by *A. acidipropionici* via sequential batches with cell recycling had considerably enhanced the volumetric rates; the PA yield, PA- and by-products concentrations were not affected, indicating the high stability of this system. However, the specific cell activity was decreased by increasing cell density, since the equivalent increase in volumetric rates was not achieved. This was obvious when calculating the specific production rates (*r*_*P*_) (Tables 1 and 2, Figs. 3D and 6D), which was likely a result of increased viscosity of the culture medium resulting from high cell mass, cell crowdedness, limited nutrients (mainly N-source and vitamins), and long-term exposure to PA and other acidic by-products [14, 43].

Compared to our previous study on PA production from glycerol (50 g/L) using the same microorganism, culture medium, and operating conditions [14], glucose yields almost two times higher cell density than glycerol (~ 40.0 g/L *vs.* 24.66 g/L), however, with lower PA yield (0.56 g_PA_/g_Glu_ *vs.* 0.79 g_PA_/g_Gly_), -volumetric productivity (1.15 g/L.h *vs.* 1.42 g/L.h), and PA:SA:AA mass ratio (100:25:22 *vs.* 100:24:9). Fermentation of the glycerol/glucose mixture (90 g/L total C-sources) was somewhat similar to that of 85 g/L glycerol with regards to cell density (20 g_CDW_/L), and PA-, SA-, and AA concentrations but with a lower PA volumetric productivity (0.48 *vs.* 0.77), [14]. The addition of glycerol as a feed after 24 h or 48 h of glucose fermentation rather than at the beginning of the fermentation was reported to enhance the yield of both PA (0.7 g/g C-sources) and vitamin B12 (0.72 mg/g C-sources) by *Propionibacterium freudenreichii* [48].

The production of PA from glycerol/glucose mixture has been investigated in several studies. For example, in one study, *A. acidipropionici* cells produced 21.9 g/L PA from ~ 31 g/L of total C-source at a rate of 0.152 g/L.h [23]. In another study, batch fermentation of ~ 31 g/L total C-sources resulted in ~ 18 g/L PA at a rate of 0.228 g/L.h [25]. The PA yield reached 0.53 g_PA_/g_C-source_, and the ratios of PA/AA and PA/SA were 13.51 g_PA_/g_AA_ and 8.17 g_PA_/g_SA_, respectively. Further cell immobilization in a fibrous-bed bioreactor (FBB) operated in repeated cycles converted ~ 36 g/L total C-source into 20 g/L PA at a maximum rate of 0.58 g/L.h with a yield of 0.58 g/g using yeast extract and tryptic soy broth as N-sources [25]. However, replacing the synthetic culture medium with crude glycerol and cassava bagasse hydrolysate as C-sources and corn steep liquor as N-source (raw materials), the PA productivity was significantly reduced to 0.25 g/L.h [25]. This productivity and PA concentration (~ 18 g/L) are almost half that obtained in the present study using HTPJ, crude glycerol (raw materials), and glucose as a culture medium.

When glucose was used as the sole C-source, the maximum specific consumption rate during the first batch reached 0.18 g_Glu_/g_X_.h and then remained relatively constant between 0.08 and 0.12 g_Glu_/g_X_.h throughout the subsequent batches (Fig. 3). When glycerol/glucose mixture was used, their consumption occurred simultaneously, however there was a slight preference towards glucose assimilation over glycerol. This was obvious during the first batch and partially observed in the latter batches. The specific glycerol/glucose assimilation rate reached a maximum of 0.25 g_S_/g_X_.h (0.11 g_Glu_/g_X_.h and 0.14 g_Gly_/g_X_.h, respectively) during the first batch and then remained between 0.10 g_Glu_/g_X_.h and 0.08 g_Gly_/g_X_.h, respectively, throughout the remaining batches (Fig. 6).

SA and AA were the main by-products obtained. These acids represented 33.3% of total acids produced in the case of glucose while representing only 25% in the case of glycerol/glucose co-fermentation. Although the percentage of SA is almost the same in both cases, AA accumulates to between 3.5 and 8.5 times higher concentrations in the case of glucose than in a glycerol/glucose mixture. AA production has been repeatedly reported as a major limitation when glucose is used as a C-source [59]. Glucose is a more oxidized C-source (degree of reduction “γ” = 4) and yields lower amounts of reducing equivalents than glycerol (γ = 4.67). Therefore, its assimilation by *A. acidipropionici* yields more acetic acid (γ = 4) as a by-product [3, 7, 13, 20, 21]. Moreover, the metabolic flux towards AA generates more ATP, which is utilized mainly in cell growth [7]. Unfortunately, these acidic by-products interfere with PA purification steps, adding to the process costs.

In an attempt to minimize acidic by-products, Wang et al. (2015) engineered *P. freudenreichii* subsp. *shermanii* by overexpressing propionyl-CoA:succinyl-CoA transferase resulting in an increased carbon flux towards PA [24]. When using glycerol/glucose mixture, a higher PA yield of 0.62 g/g along with a high productivity of 0.41 g/L.h and lower SA and AA concentrations than those of the wild-type strain were observed [24].

Supplementing the culture medium with the required vitamins and co-factors can further enhance the fermentation kinetics as reported earlier [14, 43]. Propionibacteria can synthesize different vitamins; however, biotin, thiamine, and pantothenic acid must be supplied [60]. Although potato juice is a promising N-source, it lacks biotin. The enzyme catalyzing the transfer of the carboxyl group from S-methylmalonyl-CoA to pyruvate to form oxaloacetate and propionyl-CoA requires biotin as a co-factor [55]. Earlier, it was shown that reducing the biotin and HTPJ concentrations to 0.25 mg/L (instead of 0.5 mg/L) and 0.5× (instead of 1×), respectively, in the last two batches of eleven successive batches with cell recycle, resulted in lower AA and SA production without impacting PA production [14]. However, in the present study, attempts to avoid biotin supplementation from the beginning of the batch fermentations were unsuccessful since it affected the fermentation kinetics, mainly cell growth and PA production.

## Conclusions

HCD fermentations offer smart solutions to the well-reported problem of slow Propionibacteria growth and low PA volumetric production rates. The stability of the fermentation kinetics over several sequential batches is characteristic, highlighting the industrial potential of this system. This system is more advantageous than in continuous operation with regards to process stability, operating cost, and technical operation. In order to be economically competitive, the HCD fermentations can be applied to a propionate-tolerant strain to overcome the product inhibition of the producer microorganism.

## Methods

### Chemicals

Biotin, NH_4_OH (28%), and l-cysteine HCl - anhydrous (98%) were products of Sigma-Aldrich (St. Louis, MO, USA). Glucose, potassium dihydrogen phosphate and dipotassium hydrogen phosphate were products of Merck (NJ, USA), while Bacto yeast extract was obtained from Difco (Detroit, Michigan, USA). Concentrated HTPJ (3×) was obtained from Lyckeby Starch AB (Kristianstad, Sweden), and biodiesel-derived Glycerine Tech® was a product of Perstorp AB (Perstorp, Sweden) (for chemical composition and organoleptic characteristics, see Additional file 1).

### Microorganisms and culture conditions

*Acidipropionibacterium acidipropionici* DSM 4900 was used for the production of propionic acid. The medium used for inoculum preparation contained per liter: 10 g yeast extract, 0.25 g cysteine HCl, 1.5 g KH_2_PO_4_ and 2.5 g K_2_HPO_4_ (pH 7). Glycerine Tech® 20 g/L was included in the case of fermentation using a glucose/glycerol mixture only. For inoculum preparation, the stock culture in 15% glycerol was propagated twice anaerobically in 20 mL medium placed in 30-mL serum bottles and incubated at 32 °C first for 4 and then 2 days, respectively [14].

### Bioreactor design

Propionic acid fermentation was investigated using a 1-L Biostat-Q glass bioreactor (B. Braun, Biotech International, Germany) with a 300 mL working volume. The fermentation conditions were pH 6.5 adjusted by the addition of 5 N ammonium hydroxide, and temperature 32 °C controlled via a water jacket. The stirring speed was maintained at 200 rpm via an integrated magnetic stirring unit. At the beginning of the fermentation, the medium was bubbled with oxygen-free nitrogen gas, and then the head plate was connected to a nitrogen gas bag to keep the overhead always anaerobic during the whole fermentation.

### Sequential batch fermentation

A total of 9 consecutive sequential batches with cell recycling were performed using a culture medium of 40 g/L glucose, HTPJ (1 ×) to a final volume of 300 mL, and 0.5 mg/L biotin. The first batch started with the addition of 30 mL of fresh inoculum to 300 mL medium. The subsequent batches were inoculated using recycled cells from the preceding batch. At the end of each batch, the culture was centrifuged at 15,000×*g* and 4 °C for 10 min. The supernatant was discarded, and the cell pellet was aseptically resuspended in 30 mL of culture medium that was then transferred to 270 mL medium in the bioreactor (total 300 mL medium). Consumption of the entire carbon source was chosen as the termination point for each batch.

Another set of sequential batches with cell recycling was performed for 10 consecutive cycles using a culture medium of 30 g/L glucose, 60 g/L crude glycerine Tech® and concentrated HTPJ (1.25 ×) to a final volume of 300 mL. To study the effect of biotin on the fermentation process, the culture medium was supplemented with 0.5 mg/L in the 7th batch and with 0.75 mg/L during the last 3 batches (8, 9, and 10).

### Quantitative analyses

*Cell density:* The cell density was followed by measuring the optical density at 620 nm (OD_620nm_) using a spectrophotometer (Ultrospec 1000, Pharmacia Biotech, Uppsala, Sweden) after proper dilution of the sample.

*Cell dry weight:* For measuring the cell dry weight (CDW - g/L), 10 mL of the fermentation broth was collected in a 15-mL Falcon tube, centrifuged at 5000×*g* for 10 min at room temperature. The supernatant was discarded, and the cell pellet was dried overnight at 105 °C.

$$Cell\, dry\, weight \,(mg/mL)=(weight\, of\, the \,dry \,tube \,with \,cell \,pellet-weight \,of \,the \,dry \,tube)/10$$

Finally, the optical density was correlated with the cell dry weight (g/L) where 1 OD_620nm_ unit was equivalent to 0.366 g_CDW_/L.

*Analytes:* The concentration of the substrates (glucose and glycerol) and products (PA, AA, and SA) was determined using JASCO HPLC (Tokyo, Japan) as described elsewhere [61]. Briefly, 50 µL of properly diluted and acidified samples were injected into the mobile phase (0.5 mM sulfuric acid), flowing at a rate of 0.4 mL/min. Separation of the different components was done at 55 °C using the Biorad column, Aminex HPX-87H (Richmond, CA, USA). The detection was done using an ERC refractive index (RI) detector (Kawaguchi, Japan).

### Fermentation kinetics

The addition of the base (5N NH_4_OH) for neutralizing the pH resulted in medium dilution that affected the concentration of substrate and metabolites. The concentrations of the different compounds presented in figures and tables are the actual readings determined by HPLC. However, the dilution factor was considered to calculate of volumetric productivity and yield.

- *Volumetric PA productivity: Q*_*P*_ (g/L.h) = [(*P*_final_*dilution factor) – *P*_initial_]/[*Δt*]
- *Specific PA productivity: r*_*P*_ (g_PA_/g_CDW_.h) = *Q*_*P*_/*X*
- *Volumetric biomass productivity: Q*_*X*_ (g/L.h) = [(*X*_final_*dilution factor) – *X*_initial_]/[*Δt*]
- *Volumetric consumption rate: Q*_*S*_ (g/L.h) = [(*S*_final_*dilution factor) – *S*_initial_]/[*Δt*]
- *Specific consumption rate: r*_*S*_ (g_Gly_/g_CDW_.h) = *Q*_*S*_/*X*
- *Yield: Y*_*P/S*_ (g_P_/g_S_) = [(*P*_final_*dilution factor) – *P*_*initial*_]/[(*S*_final_*dilution factor) − *S*_*initial*_]
- *Specific growth rate (µ)* = *(Ln X*_*2*_* – Ln X*_*1*_*) / (t*_*2*_* – t*_*1*_*)*
- Duplication time (*t*_*d*_) = *Ln (2)/µ*

where *P* is the product concentration, *S* is the substrate concentration, *X* is the biomass concentration and *µ* is the specific growth rate.

### Supplementary Information

**Additional file 1.** Chemical composition and organoleptic characteristics of Glycerine Tech®.

## Data Availability

The datasets used and/or analyzed during the current study are available from the corresponding author on reasonable request.
