# Supplementary material for: High cell density sequential batch fermentation for enhanced propionic acid production from glucose and glycerol/glucose mixture using Acidipropionibacterium acidipropionici
Source: Microb Cell Fact. 2024 Mar 26;23:91. doi: 10.1186/s12934-024-02366-5 (PMC10964606; doi:10.1186/s12934-024-02366-5)
Supplement: Supplementary file 1 — Additional file 1. Chemical composition and organoleptic characteristics of Glycerine Tech®. [file 12934_2024_2366_MOESM1_ESM.docx]

**Additional file:**

**Microbial Cell Factories**

**High cell density sequential batch fermentation for enhanced propionic acid production from glucose and glycerol/glucose mixture using *Acidipropionibacterium acidipropionici***

*Tarek Dishisha^a^, Mridul Jain^b^, and Rajni Hatti-Kaul^b,^**

*^a^Department of Pharmaceutical Microbiology and Immunology, Faculty of Pharmacy, Beni-Suef University, 62511 Beni-Suef, Egypt*

*^b^Division of Biotechnology, Department of Chemistry, Center for Chemistry and Chemical Engineering, P.O. Box 124, SE-221 00 Lund, Sweden*

****Corresponding author:***

Rajni.Hatti-Kaul@biotek.lu.se

**Glycerine Tech®**

**Chemical composition:**

| **Glycerine content** | Min. 97% |
| --- | --- |
| **Water content** | Max. 1% |
| **Methanol content** | Max. 0.10% |

**Organoleptic characteristics:**

| **Hydroxyl number** | 1800 mg KOH/g |
| --- | --- |
| **Colour** | 600 APHA |
| **Ash content (as sodium)** | 40 ppm |
| **Saponification value** | 6 mg KOH/g |
| **Acid value** | 2 mg KOH/g |
